# A recombinant HER2/neu expressing listeria monocytogenes (Lm-LLO) immunotherapy delays metastatic disease and prolongs overall survival in a spontaneous canine model of osteosarcoma - a Phase I clinical trial

**DOI:** 10.1186/2051-1426-2-S3-P55

**Published:** 2014-11-06

**Authors:** Josephine S Gnanandarajah, Georges Habineza Ndikuyeze, Julie B Engiles, Anu Wallecha, Nicola Mason

**Affiliations:** 1 University of Pennsylvania, Philadelphia, PA, USA; 2 University of Pennsylvania, Kennett Square, PA, USA; 3 Advaxis, Inc, Princeton, NJ, USA

## 

Osteosarcoma (OSA) is an aggressive mesenchymal bone tumor that affects ~3000 children annually in the USA. Treatment consists of chemotherapy, radiotherapy and radical surgery. Despite treatment, metastatic disease is common and results in 30-40% mortality within 5 years. Novel therapies that prevent metastatic disease are required to improve outcome. HER2/Neu is a tyrosine kinase receptor belonging to the EGFR family. It is expressed in ~40% of pediatric OSA and is linked to reduced chemotherapeutic response, high metastatic rates and short overall survival time. Recent reports indicate that HER2/Neu is expressed on OSA tumor initiating cells and that immune targeting of HER2/Neu delays metastatic disease.

Large breed dogs spontaneously develop OSA that recapitulates many aspects of pediatric OSA including histologic heterogeneity, aggressive local disease and early metastases. At diagnosis, 95% of dogs have micrometastatic disease and despite amputation and chemotherapy, the median survival time is 10 months with most dogs euthanized due to progressive metastatic disease. As in pediatric OSA, HER2/Neu is expressed in ~40% of canine appendicular OSA making dogs a relevant model to evaluate the effects of HER2/Neu targeted immune therapy on metastatic disease prevention.

We performed a Phase I clinical trial to evaluate the safety and efficacy of an attenuated, recombinant *Listeria monocytogenes *(*Lm*) expressing a chimeric human HER2/Neu fusion protein (ADXS31-164) to prevent metastatic disease in dogs with HER2/Neu+ appendicular OSA. *Lm *secretes a pore-forming lysin, listeriolysin O (LLO) that enables it to escape the phagosome and access the class I processing machinery of antigen-presenting cells. As such, recombinant *Listeria*, engineered to express tumor antigens fused to LLO, induce potent tumor-specific CD8 T cells that mediate tumor regression in murine models. Seventeen dogs with HER2/Neu+ OSA that had undergone amputation and carboplatin chemotherapy received 1 × 10^8^, 5 × 10^8^, 1 × 10^9 ^or 3 × 10^9 ^CFU of ADXS31-164 intravenously every 3 weeks for three administrations. ADXS31-164-associated toxicities were low grade and transient. Treated dogs failed to develop pulmonary metastatic disease and showed a statistically significant increase in overall survival compared to a historical HER2/Neu+ control group. 14/17 treated dogs are still alive; median survival in HER2/Neu+ control dogs (n = 13) was 316 days (p = 0.032). ELISpot assays are underway to evaluate ADXS31-164-associated HER2/Neu specific immune responses. Our results indicate that ADXS31-164 significantly delays metastatic disease in a clinically relevant, spontaneous model and have important implications for pediatric OSA.

